# Population Genetic Analysis Reveals a High Genetic Diversity in the Brazilian *Cryptococcus gattii* VGII Population and Shifts the Global Origin from the Amazon Rainforest to the Semi-arid Desert in the Northeast of Brazil

**DOI:** 10.1371/journal.pntd.0004885

**Published:** 2016-08-16

**Authors:** Ana C. P. Souto, Lucas X. Bonfietti, Kennio Ferreira-Paim, Luciana Trilles, Marilena Martins, Marcelo Ribeiro-Alves, Cau D. Pham, Liline Martins, Wallace dos Santos, Marilene Chang, Fabio Brito-Santos, Dayane C. S. Santos, Silvana Fortes, Shawn R. Lockhart, Bodo Wanke, Márcia S. C. Melhem, Márcia S. Lazéra, Wieland Meyer

**Affiliations:** 1 Evandro Chagas National Institute of Infectious Diseases, Oswaldo Cruz Foundation, Rio de Janeiro, Brazil; 2 Institute Adolfo Lutz, São Paulo, Brazil; 3 Molecular Mycology Research Laboratory, Centre for Infectious Diseases and Microbiology, Marie Bashir Institute for Emerging Infectious Diseases and Biosecurity, Sydney Medical School-Westmead Hospital, The University of Sydney, The Westmead Institute for Medical Research, Sydney, Australia; 4 Infectious Disease Department, Triângulo Mineiro Federal University, Uberaba, Brazil; 5 Centers for Disease Control and Prevention, Atlanta, United States of America; 6 University of Piauí State, Teresina, Brazil; 7 Federal University of Pará, Belém, Brazil; 8 Federal University of Mato Grosso do Sul, Campo Grande, Brazil; 9 Biodiversity Research Center, Federal University of Roraima, Boa Vista, Brazil; University of Tennessee, UNITED STATES

## Abstract

*Cryptococcus neoformans* and *Cryptococcus gattii* are responsible globally for almost one million cryptococcosis cases yearly, mostly in immunocompromised patients, such as those living with HIV. Infections due to *C*. *gattii* have mainly been described in tropical and subtropical regions, but its adaptation to temperate regions was crucial in the species evolution and highlighted the importance of this pathogenic yeast in the context of disease. *Cryptococcus gattii* molecular type VGII has come to the forefront in connection with an on-going emergence in the Pacific North West of North America. Taking into account that previous work pointed towards South America as an origin of this species, the present work aimed to assess the genetic diversity within the Brazilian *C*. *gattii* VGII population in order to gain new insights into its origin and global dispersal from the South American continent using the ISHAM consensus MLST typing scheme. Our results corroborate the finding that the Brazilian *C*. *gattii* VGII population is highly diverse. The diversity is likely due to recombination generated from sexual reproduction, as evidenced by the presence of both mating types in clinical and environmental samples. The data presented herein strongly supports the emergence of highly virulent strains from ancestors in the Northern regions of Brazil, Amazonia and the Northeast. Numerous genotypes represent a link between Brazil and other parts of the world reinforcing South America as the most likely origin of the *C*. *gattii* VGII subtypes and their subsequent global spread, including their dispersal into North America, where they caused a major emergence.

## Author Summary

*Cryptococcus neoformans* and *Cryptococcus gattii* are fungal agents responsible globally for almost one million cryptococcosis cases yearly, mostly in immunocompromised patients, such as those living with HIV. Cryptococcosis is a life-threatening mycosis, frequently causing meningoencephalitis. Infections due to *C*. *gattii* were originally described in tropical and subtropical regions, but its adaptation to temperate regions was highlighted by the emergence in the Pacific North West of North America by *C*. *gattii* molecular type VGII. The present work aimed to assess the genetic diversity within the Brazilian *C*. *gattii* VGII population to gain new insights into its origin and global dispersal from the South American continent using the ISHAM MLST consensus typing scheme. Our results corroborate that the Brazilian *C*. *gattii* VGII population is highly diverse, and strongly supports the emergence of highly virulent strains from ancestors in the Northern regions of Brazil. Numerous genotypes represent a link between Brazil and other parts of the world, and the isolates from the transitional ecological area in Northeast Brazil are the most likely ancestor lineages, translocating from caatinga/cerrado by adapting progressively throughout Amazonia in South America, and spread to the North American Pacific Northwest region and other parts of the world on multiple occasions.

## Introduction

Cryptococcosis is a life-threatening mycosis with high lethality rates, especially in underdeveloped countries [1]. Infection occurs via the respiratory route by inhalation of infectious propagules (desiccated yeast cells or basidiospores) of *Cryptococcus neoformans* and *C*. *gattii*, frequently spreading to the central nervous system causing meningoencephalitis, with a lethality rate of up to 70% within three months after diagnosis [1, 2]. *C*. *neoformans* is a cosmopolitan and primarily opportunistic agent, comprising the major molecular types VNI, VNII (VNB), VNIII and VNIV. By contrast, *C*. *gattii* infects mainly otherwise immunocompetent hosts, although a previous study suggests that some immune profile deficiency not detected by routine tests may predispose immunocompetent individuals to meningoencephalitis by *C*. *gattii* [3]. Besides the well-known outbreak in North America, *C*. *gattii* infections occur in large areas of the Amazon region and in the semi-arid Northeast region of Brazil [4, 5, 6, 7], being the major molecular types VGI, VGII, VGIII and VGIV. The molecular types of both species have been recently described as new species [8]. To enable a clear connection to previous published work this report maintains the two species concept with its molecular type-based nomenclature.

*C*. *gattii* VGI and VGIII had been the primary cause of human and animal infections until 1999 in North America, when isolates of the molecular type VGII were reported as the cause of an outbreak affecting hundreds of healthy humans and animals in British Columbia, Canada. This outbreak lineage subsequently spread to the Pacific Northwest (PNW) of the USA in the following years [9]. Alternatively, based on one clinical case reported from the 1970s, which described a VGII isolate NIH444 from Seattle (USA), it could be suggested that the VGII outbreak lineage was already present in the temperate region several decades before its emergence on Vancouver Island [10]. However, the genotype of this isolate is very different from the Vancouver Island outbreak lineages [11] making in unlikely to be the source of the Vancouver Island outbreak.

Later on, PCR-fingerprinting, Amplification Fragment Length Polymorphism (AFLP) analysis and Multilocus Sequence Typing (MLST) identified three distinct clonal lineages (subtypes) responsible for the majority of cases in the PNW [9, 12]: VGIIa, the most common genotype, VGIIb, the less common [13], and VGIIc, a subsequently identified genotype with a confined geographic distribution [12]. Following this, the ISHAM working group of the International Society for Human and Animal Mycology (ISHAM) on genotyping of *C*. *neoformans* and *C*. *gattii* proposed a standardized MLST scheme, using six housekeeping genes and the IGS1 region as method of choice for strain subtyping to obtain comparable subtyping results worldwide [14]. MLST confirmed the same three major genotypes within North America [11, 15].

The emergence of infections by *C*. *gattii* VGII in temperate regions initiated a pursuit of the origin of the Vancouver Island outbreak strains. One hypothesis is the occurrence of same-sex mating from an Australasian population, giving rise to a virulent genotype, which was subsequently dispersed [16, 17]. However, a study using coalescence gene genealogy, phylogenetic and recombination analysis suggested that it may alternatively have emerged from a highly-recombining *C*. *gattii* population in the native rainforest of Northern Brazil, subsequently dispersed out of the original tropical area, reaching North America [18]. Similarly, two recent population genetic analyses using Single Nucleotide Polymorphism (SNP) analysis based on whole genome sequence data provided additional evidence that the PNW strains originated from South America [11, 16].

Based on the above mentioned findings the present work aimed to assess the genetic variability within the Brazilian VGII population and to gain new insights related to the population structure, its origin and global dispersal from the South American continent.

## Methods

### Isolates

One hundred and forty five Brazilian clinical and environmental isolates of the major *C*. *gattii* molecular type VGII identified by *URA5*-RFLP analysis [19] stored in the Culture Collection of Pathogenic Fungi, at the Oswaldo Cruz Foundation, Rio de Janeiro, and in the Research Collection of the Adolf Lutz Institute, São Paulo, Brazil were studied retrospectively. In addition to the Brazilian isolates, 42 published sequence types (STs) from Brazil and other countries, representing all previously published VGII sequence types, maintained in the MLST database (mlst.mycologylab.org), were used for comparison, in order to place the Brazilian population in an international context. For isolate information, see S1 Table.

### MultiLocus Sequence Typing (MLST)

The molecular subtypes and the genetic diversity of the Brazilian *C*. *gattii* VGII isolates were investigated using the ISHAM MLST consensus scheme for *C*. *neoformans* and *C*. *gattii* [14]. Seven unlinked genetic loci were amplified, including the genes *CAP59*, *GPD1*, *LAC1*, *PLB1*, *SOD1* and *URA5* and the IGS1 region, using the published PCR conditions for all seven loci [14]. The sequences were manually edited using the software Sequencher 5.3 (Gene Codes Corporation, MI, USA) and aligned using MEGA 6.06 [20]. The allele types and the sequence types (ST) were identified via sequence alignments against the *C*. *gattii* MLST database available at http://mlst.mycologylab.org/. The sequences of all newly identified allele types have been submitted to the *C*. *gattii* MLST database and GenBank.

### Phylogenetic analyses

In order to infer the phylogenetic relationships of the isolates, the best evolutionary model for concatenated sequences of the seven loci was selected using the software jModelTest 2.1.7 [21, 22] applying the corrected Akaike Information Criterion (AIC) and/or Bayesian information criteria (BIC). The model K80 + I + G with Ti/Tv: 3.4548 and gamma shape 0.4430 [23] was the best model for the concatenated dataset, which was then used in the software MEGA 6.06 [24] to construct an unrooted Maximum Likelihood (ML) phylogenetic tree. In addition, the dataset was submitted to Neighbour Joining (NJ) analysis based on the K80 [23] model and Maximum Parsimony (MP) based on the nucleotide substitution model and using the Subtree-Pruning-Regrafting (SPR) algorithm [11]. For the ML and MP methods, all sites were included in the analysis while for NJ, all positions containing alignment gaps were eliminated. Bootstrap analysis using 1,000 replicates was used to estimate support for the identified clades of the concatenate dataset in all analysis.

The minimum spanning tree using the goeBURST algorithm in the PHILOVIZ software (http://www.phyloviz.net/wiki/) [25] was generated from concatenated sequence regions to visualize the relatedness of the *C*. *gattii* isolates with their region of origin. The diagrams show where the ST differs in the single locus variant (SLV), double locus variant (DLV), and triple locus variant (TLV), respectively. A clonal complex (CC) concept was adopted when a SLV linkage with the founder ST was found [24, 25].

### Population structure

In order to better understand the correct number of *C*. *gattii* VGII populations (*K*) that were geographically homogeneous and maximally differentiated from each other, and to evaluate the presence of immigrant individuals with respect to their geographical population, we used a Bayesian statistical model [26], which calculates the membership coefficient to each of the population using the software STRUCTURE 2.3.4, available at http://pritchardlab.stanford.edu/structure.html [27]. Twenty runs were performed for each value of the number of populations (*K*) ranging from 1 to 10. Each run consisted of Markov-chain Monte Carlo (MCMC) simulations of 1,000,000 interactions with a burn-in period of 100,000 generations. The model selected was Admixture model that takes into account the presence of migrants in the population. The actual number of *K* was calculated using the average and standard deviation of each *K* using the *ad hoc* statistic of the software Structure Harvester available at http://taylor0.biology.ucla.edu/structureHarvester/ [28]. The results of the coefficients of the optimal *K* were graphed using the software Clumpp version 1.1.2 available at https://web.stanford.edu/group/rosenberglab/clumpp.html [29] and Structure plot [30].

### Recombination, clonality and nucleotide diversity analysis

The software DnaSP 5.10 (http://www.ub.edu/dnasp/) [31] was used to analyse the haplotype diversity (Hd) and nucleotide diversities. The presence of recombination in the dataset was checked by phylogenetic compatibilities of nearby polymorphic sites along single and concatenated sequences in the software SplitsTree v. 4.13.1 (http://www.splitstree.org/) [32]. Recombination events can be visualized by the formation of parallelograms between the neighbours using the reticulated algorithm NeighborNet. The Pairwise Homoplasy Index (PHY) test implemented in SplitsTree v. 4.13.1 and the pairwise linkage disequilibrium (D) available in the software DnaSP v. 5.10 were also used to detect the presence of recombination. To perform the recombination analysis, the optimal molecular evolutionary model per gene was selected in the software jModelTest 2.1.7 as described above for the phylogenetic analysis and applied in the software SplitsTree v. 4.13.1. Thus, the parameters were used as follows: *CAP59*: K80 + I, Ti/Tv: 31.3584, and pinv: 0.9740; *GPD1*: K80, Ti/Tv: 2.7548; IGS1: F81; FA (0.2655), FC (0.1533), FG (0.2906), FT (0.2903); *LAC1*: K80, Ti/Tv: 3.0044; *SOD1*: K80 + I + G, Ti/Tv: 1.8129, pinv: 0.9360, and alpha: 0.7400; *URA5*: JC; *PLB1*: F81, FA (0.2336), FC (0.2036), FG (0.2908), and FT (0.2725).

The standard index of association (*I*_A_) is a measure of linkage disequilibrium of genotypes and/or population [33]. This test checks the null hypothesis of linkage equilibrium and *p* <0.05 indicates that the null hypotheses of linkage equilibrium should be rejected, which means that the population is under clonal reproduction. In this study we applied also the standardized *I*_*A*_ (*I*_*A*_^*S*^) with 10,000 randomizations available in the program LIAN 3.5 (http://guanine.evolbio.mpg.de/cgi-bin/lian/lian.cgi.pl) using both the parametric method and the Monte Carlo simulation for the concatenated dataset to infer the presence of linkage disequilibrium.

### Mating typing

The mating type was characterized by PCR of the pheromone genes using primers specific for MATalpha, MFalfaU (5’TTCACTGCCATCTTCACCACC 3’) in combination with MFalfaL (5’TCTAGGCGATGACACAAAGGG 3’); and for MAT**a** JOHE9787 (5’ ACACCGCCTGTTACAATGGAC 3’) in combination with JOHE9788 (5’ CAGCGTTTGAAGATGGACTTT 3’) [34]. Amplifications of the pheromone genes MATalpha and MAT**a** were performed independently, in a final volume of 50μL containing 50 ng of DNA, 1X PCR buffer [200 mM Tris-HCl (pH 8.4), 500 mM KCl—Invitrogen], 0.2 mM each of dATP, dCTP, dGTP, and dTTP (Invitrogen), 2 mM magnesium cloride, 2.5 U Taq DNA polymerase (Invitrogen), and 50 ng of each primer. The amplification was carried out in a thermocycler (Eppendorf mastercycler gradient, California, USA) at 95°C for 3-min initial denaturation, 30 cycles at 94°C for 1 min, annealing at 57.5°C for 1 min, extension at 72°C for 1 min, and a final extension at 72°C for 7 min. The unique fragment corresponding to each mating type was visualized after 3% agarose gel electrophoresis at 100 V.

## Results

### Genetic variability and regional distribution in Brazil

A total of 145 *C*. *gattii* VGII isolates, including 127 clinical and 18 environmental isolates, collected between 1989 and 2010 in 4 out of the 5 Brazilian regions: 1. Northeast (n = 39), including isolates from Piauí (PI) and Bahia (BA); 2. North (n = 38), including isolates from Pará (PA), Amazonas (AM) and Roraima (RR); 3. Southeast (n = 59), including isolates from Rio de Janeiro (RJ) and São Paulo (SP); and 4. Central-West (n = 9), including isolates from Mato Grosso do Sul (MS). No VGII isolates were collected in the South region (Rio Grande do Sul, Santa Catarina and Paraná) of the country. The individual isolate data are in S1 Table.

MLST analysis identified 24 allele types for the *CAP59* locus, 13 for *GPD1*, nine for *LAC1*, 11 for *PLB1*, 38 for *SOD1*, eight for *URA5* and 34 for the IGS1 region. Based on the combined analysis of the seven loci, a total of 81 sequence types were observed (Table 1, S1 Table), with 100 polymorphic sites detected in 4,186 sites analysed. The haplotype diversity (Hd) of all strains was equal to 0.978, revealing a high genetic variability among the Brazilian *C*. *gattii* VGII strains. All Brazilian regions showed high haplotype diversity, with the highest one found in the Northeast (NE) region (Hd = 0.981) with 31 STs, and the lowest one in the Central-West (CW) region (Hd = 0.889) with five STs (Table 1).

**Table 1:** Characteristics of the studied Brazilian regions.

Region (studied States*)	# of Isolates	# of Sequence Types	# of Polymorphic Sites	Haplotype Diversity (Hd)	Nucleotide Diversity (π)
**Central-West** (MS)	9	5	34	0.889	0.00353
**Northeast** (PI, BA)	39	31	68	0.981	0.00328
**North** (AM, PA, RR)	38	20	55	0.905	0.00296
**Southeast** (RJ, SP)	59	33	68	0.944	0.00334
Total	145	81^¶^	100^¶^	0.978	0.00349

* MS, Mato Grosso do Sul; PI, Piauí; BA, Bahia; AM, Amazonas; PA, Pará; RR, Roraima; RJ, Rio de Janeiro; SP, São Paulo.

^¶^ The repeated sequence types from different regions are not included in the total number.

**Fig 1:**
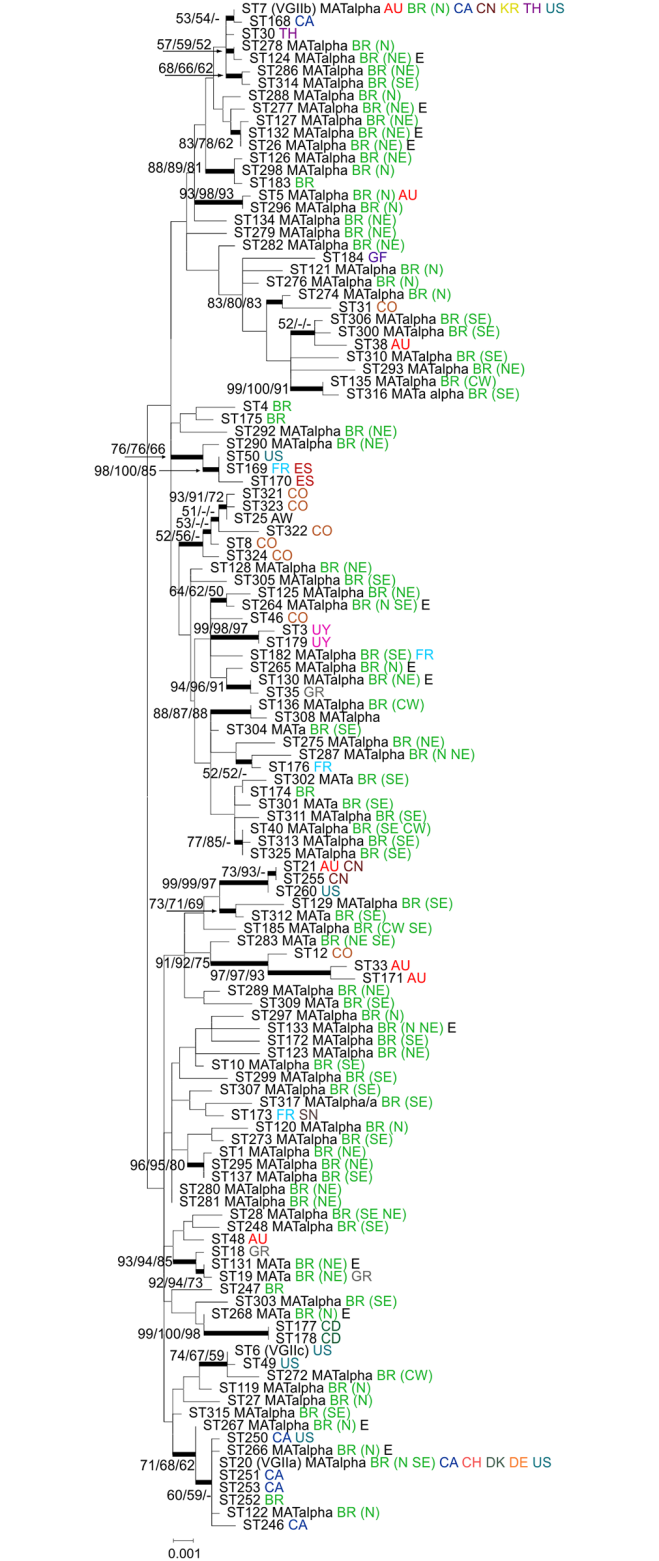
Phylogenetic analysis of Brazilian *Cryptococcus gattii* VGII isolates inferred by maximum likelihood (ML), neighbour-joining (NJ), and maximum parsimony (MP) methods using the concatenated data set of the seven MLST genes. All Brazilian Sequence types (81) from this study and 42 additional Sequence types representing all previously published VGII sequence types maintained in the MLST database (mlst.mycologylab.org) were included in the analysis. The tree with the highest log likelihood (-9194.7663) is shown. A discrete Gamma distribution was used to model evolutionary rate differences among sites (5 categories [+G, parameter = 0.0500]). The rate variation model allowed for some sites to be evolutionarily invariable ([+I], 6.6983% sites). The tree is drawn to scale, with branch lengths measuring the number of substitutions per site. Codon positions included were 1st+2nd+3rd+Noncoding. There were a total of 4,172 positions in the final dataset. Numbers at each branch indicate bootstrap values >50% based on 1,000 replicates by each of the three (ML/NJ/MP) algorithms which presented similar topologies. The taxa nomenclature includes the sequence type number (ST), Mating Type (**a** or alpha), country of isolation and for those isolates from Brazil the region of isolation [N = North, NE = Northeast, CW = Central-West, and SE = Southeast], and source (E = environmental; all others are clinical). All country abbreviations are designated according to the alpha-2 code of ISO 3166–1. AU: Australia, BR: Brazil, CA: Canada, CN: China, CO: Colombia, CD: Democratic Republic of the Congo, CH: Switzerland, DK: Denmark, DE: Germany, ES: Spain, FR: France, GF: French Guiana, GR: Greece, KR: Republic of Korea, SN: Senegal, TH: Thailand, US: United States of America, UY: Uruguay.

The genetic relationships of the obtained MLST genotypes may be separated into two main groups, the first one with 35 STs, including the VGIIa sub-genotype (ST20, major outbreak genotype on Vancouver Island), and the second one with 46 STs, including the VGIIb sub-genotype (ST7, globally present and minor outbreak genotype on Vancouver Island). No isolates of the third North American sub-genotype VGIIc (ST6) were identified in Brazil, but a closely related sequence type ST272 (strain 438BP) from MS, the CW region of Brazil was identified (Fig 1).

**Fig 2:**
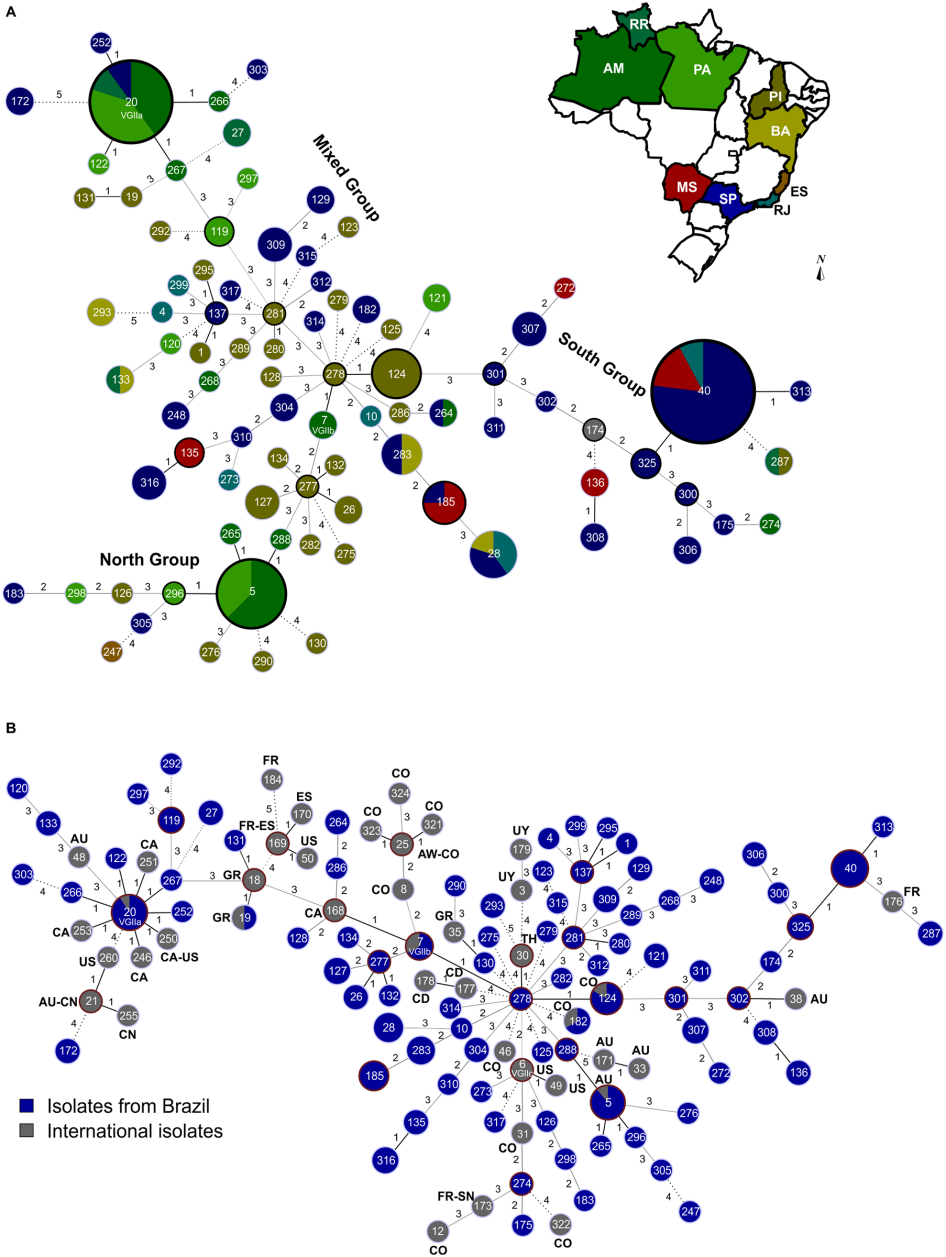
Minimum spanning tree using the goeBURST algorithm. **(A)** Minimum spanning tree using the goeBURST algorithm showing the high diversity identified among the 145 Brazilian *Cryptococcus gattii* VGII isolates and nine Brazilian sequence types (ST) according to the state where they were recovered. Dividing the country in two macro regions such as North (composing of the States of Amazonas, Roraima, Pará, Piauí, and Bahia) and South (States of Mato Grosso do Sul, São Paulo, and Rio de Janeiro) three main groups can be identified: 1) isolates mainly recovered from the South, representing all isolates derived from the ST301; 2) isolates mainly recovered from the North, representing those originated from the ST7 (VGIIb), and 3) the mixed group, which contains isolates derived from ST281. The main clonal complexes (CC) in each of these groups are: CC40, CC5, and CC20. The ancestors of the CC is highlighted by a black line. **(B)** Minimum spanning tree using the goeBURST algorithm of the isolates presented in (**A)**, and their comparison with 42 STs identified in different countries previously published. All country abbreviations are designated according to the alpha-2 code of ISO 3166–1. In both figures each circle represents a unique ST, and the circumference is proportional to the number of isolates within each ST. Solid, grey and dashed branches represent at least one, two to three, and more than four or five differences, respectively. All STs are different VGII lineages, only the three PNW outbreak genotypes are labelled specifically as VGIIa (ST20), VGIIb (ST7), and VGIIc (ST6).

Among the 81 sequence types identified in Brazil, 54 are represented by a single isolate. The most frequent subtype, ST40, accounts for 13 isolates found in the Central-West (CW) and Southeast (SE) regions, followed by ST20 (VGIIa) and ST5, which contained nine and seven isolates, respectively (S1 Table). In general, the different regions harboured different genotypes. The majority of the sequence types (n = 73) are unique for each of the Brazilian regions analysed. Only six sequence types were identified in more than one region (ST20, ST28, ST40, ST133, ST185 and ST287) (S1 Table, Fig 2A).

The regional distribution of the STs within the regions was also evaluated with the goeBURST analysis including all 145 *C*. *gattii* isolates from this study, and another nine different Brazilian STs obtained from previously published data (S1 Table, Fig 2A). In this analysis, 9 clonal complexes (CC) were identified (e.g. 9 groups presenting SLV). Clonal complex CC 278 is composed of the clinical sequence type ST278, isolated from a patient in Piauí, ST7 isolated from clinical and environmental samples from Amazonia, and ST124, isolated from clinical and environmental samples from Piauí state. ST278 seems to play an important role in the epidemiological distribution of *C*. *gattii* due to its link with the less virulent ST7 (VGIIb). In addition, three main groups are linked to CC 278: 1) ST301 and all its descendants, mainly present in the SE region of Brazil, which is a triple-locus variant (TLV) (IGS1, *PLB1*, *URA5* allele) of ST124; 2) ST277 and all its descendants, mainly present in the North (N) region of Brazil, which is a double-locus variant (DLV) (*GPD1*, *PLB1*) of ST7 (VGIIb); and 3) ST281 and all its descendants, a mixed group of strains from all regions of the country, which is a TLV (IGS1, *PLB1*, *SOD1* allele) of ST278. The important role played by ST278 isolated from the semi-arid NE region was confirmed after addition of 34 STs from other countries (Fig 2B).

Within the above mentioned three main groups, some representative clonal complexes can be identified: Clonal complex 40, composed of the ancestor ST40, which is the dominant ST in the SE and CW regions of Brazil, isolated from 13 clinical samples from São Paulo, Rio de Janeiro, and Mato Grosso do Sul, and its two single-locus variants (SLV) (ST325 and ST313), both isolated from clinical samples. Clonal complex 5 is represented by the ancestor ST5 and constituted of eight clinical and environmental isolates from the North of Brazil. The other three SLVs of ST5 are ST265, ST288, and ST296, all isolated from clinical and environmental samples. Clonal complex 20 is represented by five STs (ST20, ST122, ST252, ST266 and ST267), being the ST20 (VGIIa) the founder ST of this complex and composed by isolates from the SE and N (Fig 2A).

### Population structure

**Fig 3:**
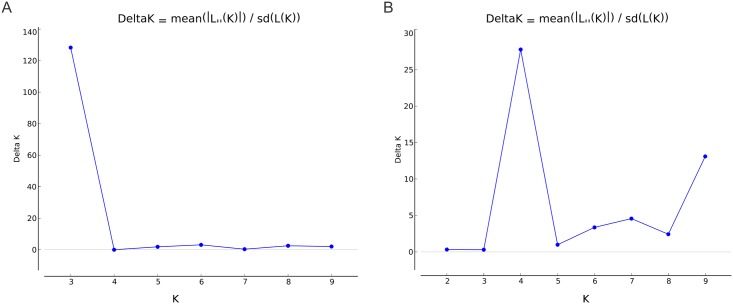
Number of populations using in the STRUCTURE analysis calculated according to [35]. The results presenting in (A) show three populations in the Brazilian *Cryptococcus gattii* VGII and in (B) show four populations in the STs identified in different countries previously published and in Brazil.

**Fig 4:**
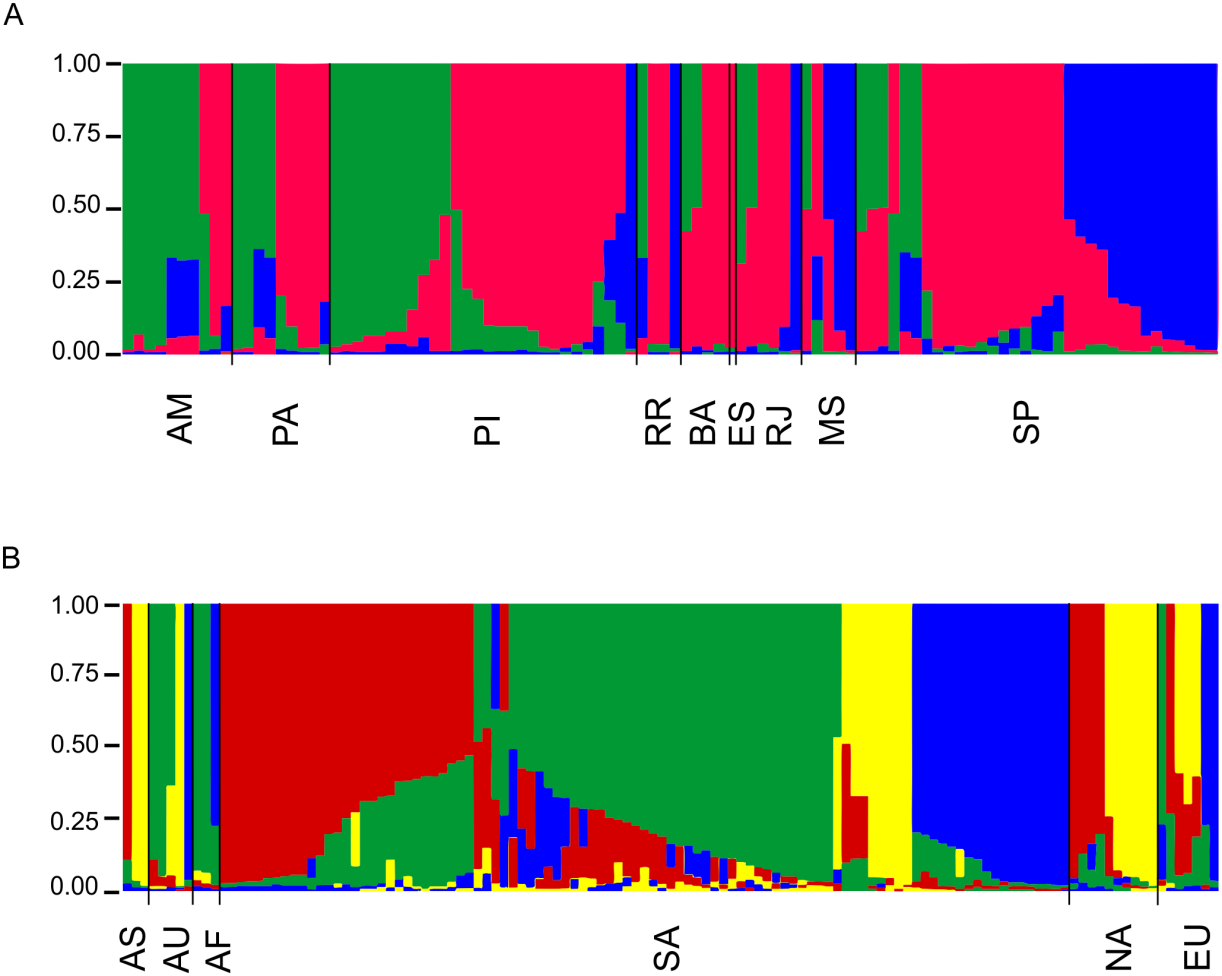
Population structure analysis inferred using multilocus sequence genotypes of *Cryptococcus gattii* isolates recovered from Brazil and using *K* = 3 (A), and comparison of these isolates with isolates recovered from different continents using *K* = 4 (B). Clusters of individuals based on prior-defined populations are referred according to the Brazilian States and/or Continent. Each vertical line represents one of the isolates included and each color (red, dark blue, dark green, and yellow) represents the most likely ancestry of each isolate from one of the three populations (A) or four populations (B). Individuals with multiple colours have admixed genotypes from the prior-defined populations. One clone per region was included, thus Figure A contains 100 isolates while Figure B contain 125 isolates. The taxa nomenclature includes AM: Amazonas, PA: Pará, PI: Piauí, RR: Roraima, BA: Bahia, ES: Espírito Santo, RJ: Rio de Janeiro, MS: Mato Grosso do Sul, AS: Asia, AU: Australia, AF: Africa, SA: South America, NA: North America, EU: Europe.

In order to better understand the number of populations and their distribution throughout the country, we applied the admixture model of Structure in our dataset and identified *K* = 3 populations (Fig 3A). A high proportion of admixture was observed in our sample (Fig 4A). One of the populations, here presented in green, was mainly found in those States from the N/NE part of the country while the population described in blue was mainly presented in the States of the SE/CW part of the country, such as São Paulo and Mato Grosso do Sul. The third population, presented in red was found to be distributed all over the country and seems to act as an important contributor of genetic material to the remaining populations. We then compared the 87 Brazilian isolates included in the 97 South American isolates, representing all STs obtained in Brazil, with isolates recovered from different regions of the world in order to see how the Brazilian population contributed to the global *C*. *gattii* VGII distribution, detecting K = 4 number of populations (Fig 3B). In this analysis, a high proportion of admixture was also detected within the whole population of *C*. *gattii* VGII and among the South American isolates one more population was detected (here identified in yellow), which mainly derived from North America, Asia and Australia (Fig 4B).

### Mating type and multilocus linkage disequilibrium

The majority of the isolates (129/145 = 89%) were identified as mating type alpha, 10% (15/145) were mating type **a**, including 13 clinical and two environmental isolates, and one isolate of clinical origin was mating type alpha/**a** (S1 Table).

**Fig 5:**
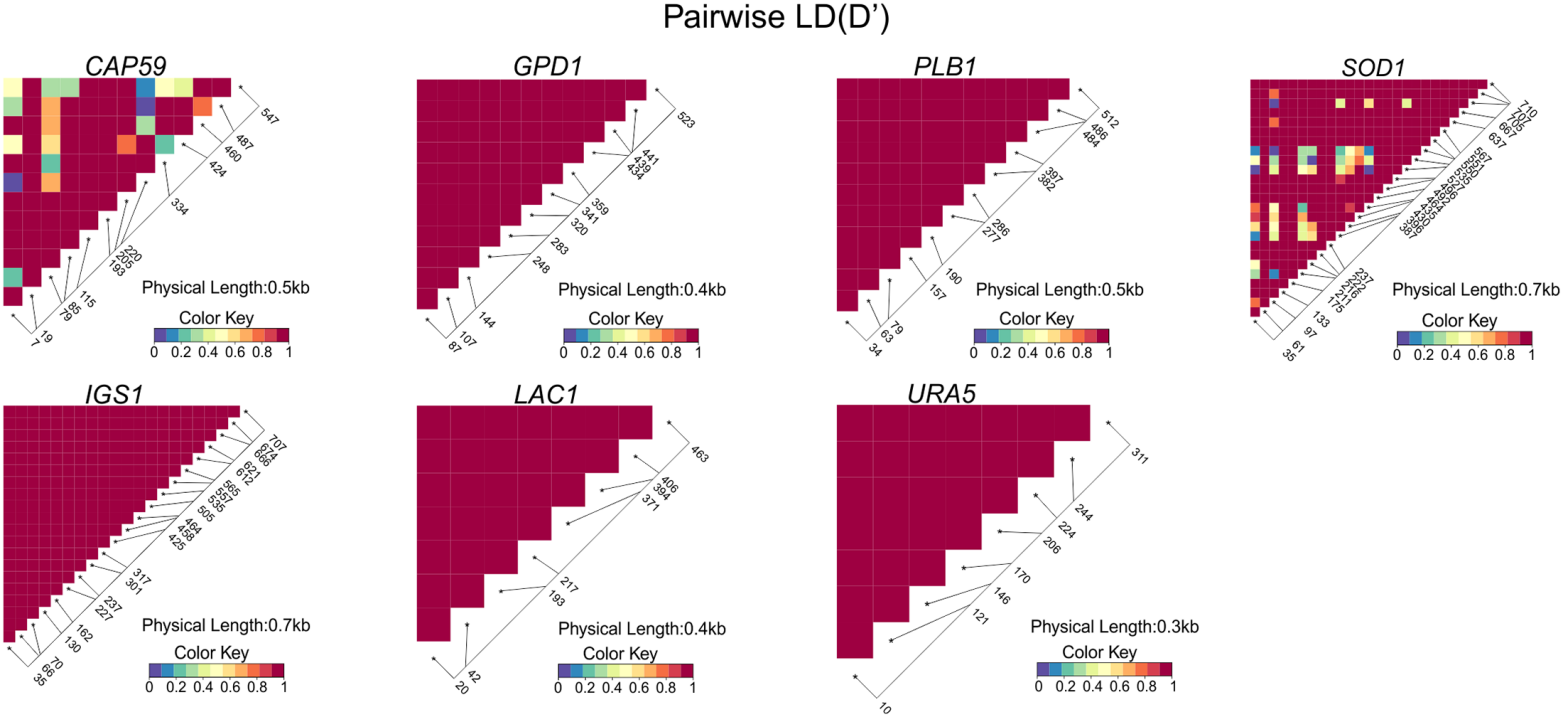
Linkage disequilibrium heat maps between polymorphic sites for all studied MLST loci (*CAP59*, *GPD1*, *PLB1*, *SOD1*, IGS1, *LAC1* and *URA5*). Pairwise D’ metricises are represented by heat colours (Colour Key). Recombination amongst Brazilian VGII strains has been shown, as evidenced amongst four sites (0.7%) in the locus *CAP59* and six sites (0.8%) in the locus *SOD1* (Fisher’s Exact Test P-value ≤ 0.05).

Random mating can be evidenced by the linkage disequilibrium (D), converging to zero. The SNPs present in the seven loci were used to detect the evidence of recombination separately. Pairwise Linkage Disequilibrium (D) between SNPs suggested at least six recombination events responsible for the polymorphism at the *SOD1* locus and four at the *CAP59* locus (D’ < 0.2) (Fig 5). The other five loci showed alleles in total disequilibrium (D’ = 1). The Brazilian VGII isolates also showed evidence of recombination with a high degree of homoplasy demonstrated by a Consistency Index (CI) of 0.27 (p<0.05) (Table 2).

**Table 2:** Characteristics of the MLST Loci studied in 145 Brazilian *C*. *gattii* VGII isolates.

Locus	Length	Polymorphic sites	Recombining position	phi test
*CAP59*	557	13	(7,79), (79,220), (334,424), (460,487)	0.005
*GPD1*	550	12	-	1.0
IGS1	717	21	-	1.0
*LAC1*	475	8	-	1.0
*PLB1*	535	12	-	0.236
*SOD1*	713	27	(35,97), (97,211), (216,387), (396,435), (435,527), (527,705)	0.049
*URA5*	638	8	-	1.0

**Fig 6:**
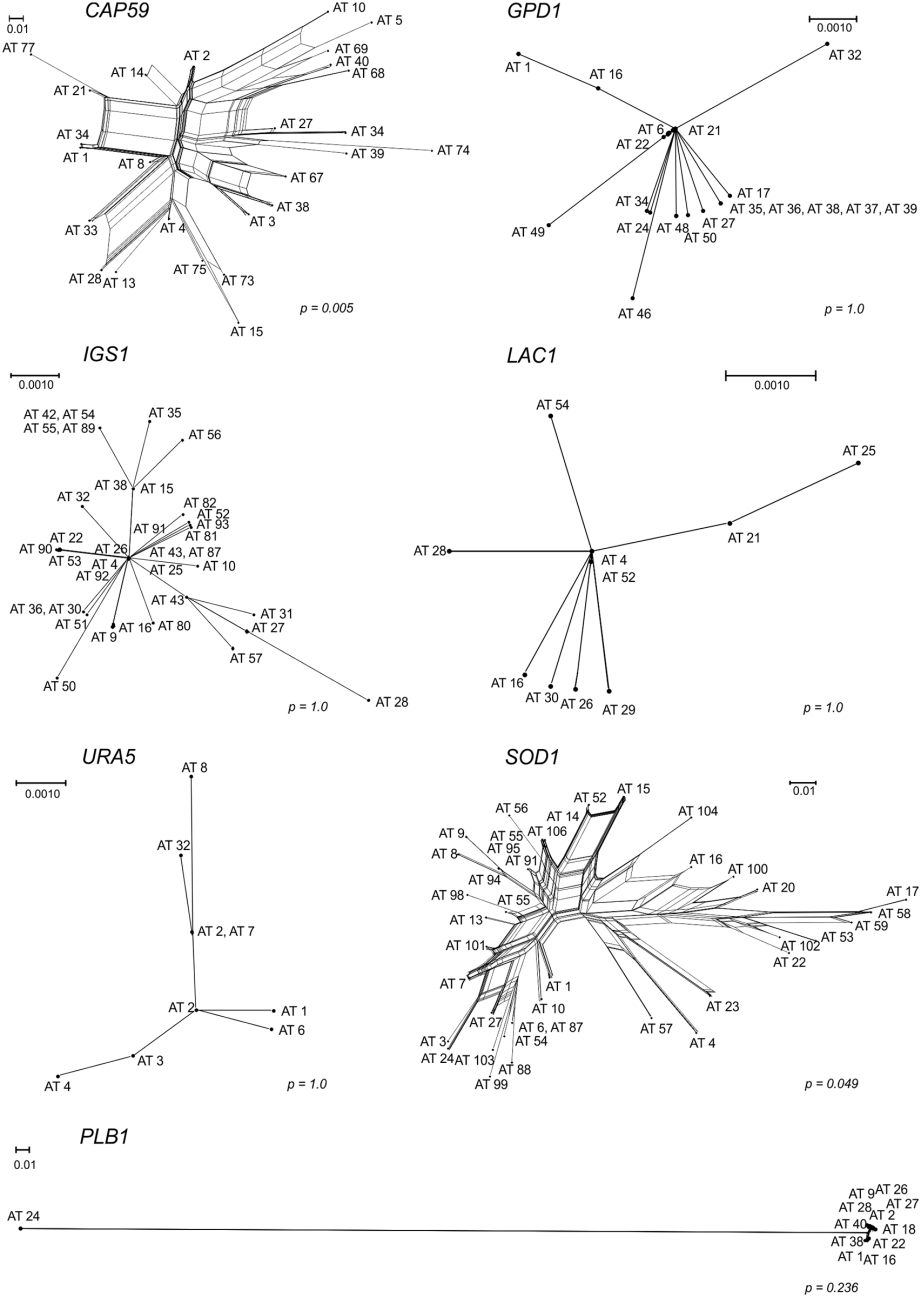
Split decomposition analysis using the Neighbor-net algorithm of the each of seven MLST genes evidencing the diversity and branching ambiguities attributable to recombination events in the *CAP59* and *SOD1*. The phi test result implemented in the software SplitsTree is presented next to each allele.

In order to confirm these results we applied two other recombination tests to our dataset. The strong reticulation in the networks and phi test implemented in the SplitsTree software for the single sequences also indicate recombination within the Brazilian VGII isolates for *CAP59* and *SOD1* (p<0.05) (Fig 6). These results were confirmed in the concatenated data set with an *I*_*A*_^*S*^ value of 0.0407 and statistically significant for recombination (p<0.0001) in the Brazilian population.

## Discussion

Since the unexpected emergence of cryptococcosis caused by the VGII subtype of *C*. *gattii* in temperate North America in 1999, it has been recognized as a major agent of severe pulmonary and neurological infections in this region [reviewed in 36]. The North American cases of human and animal cryptococcosis caused by distinct highly clonal populations (VGIIa, VGIIb and VGIIc) [11, 16] point to their capacity to emerge from original habitats to adapt and colonize new environments and hosts, rapidly multiplying the new adapted populations.

The current study shows a high genetic variability amongst Brazilian *C*. *gattii* VGII isolates, presenting 81 MLST STs in 145 clinical and environmental isolates. In addition, a high level of haplotype diversity was observed, while also demonstrating a high degree of homoplasy, with the Consistency Index suggesting the absence of a selective genetic pressure. The patterns of the polymorphisms identified among the Brazilian strains surveyed in this study indicated a history of recombination for the genetic loci *CAP59* and *SOD1* (Figs 5 and 6, Table 2), which contributed to the haplotype diversity observed. The fact that both mating types were present among the clinical and environmental Brazilian VGII isolates, with 10% of them being mating type **a**, emphasizes that recombination events are likely to occur in Brazil, leading to the great variability/high genetic diversity observed. These findings are also reinforced by the mosaic of multiple small chromosomal chunks presented in most of the isolates studied (Fig 4). Recombination amongst VGII genotypes has also been detected previously at global [15, 37] and local [38, 39] scales. Although limited number of isolates have been analysed and a very limited number of sequence types have been identified [13, 39, 40], they already indicated the occurrence of high molecular polymorphisms in South American *Cryptococcus* strains.

Despite the high genetic diversity in the Brazilian *C*. *gattii* VGII population, nine clonal complexes were found. Some are represented by very common and frequently recovered STs in clinical and environmental samples (e.g. ST20-VGIIa, ST40, and ST5). The persistence of successful STs, which are stable in space and time and most significant in cases of widespread adapted clones, may follow the features of clonal evolution which is defined as strongly restrained recombination [41]. This has been described for several microorganisms, in bacteria [42], protozoa [43], and fungi [44]. Linked populations have been identified as most likely being stepping stones in the global spread of VGII. Analysis of VGII in Australia [39] identified six sequence types (ST7 (VGIIb), ST38, ST5, ST21, ST33 and ST48), suggesting an introduction into Australia, which created a possible founder effect followed by a clonal expansion of the subtypes. In Thailand, the majority of the *C*. *gattii* isolates belonged to the sub-genotype VGIIb (11 out of 12) [45], suggesting again a clonal expansion of this subtype.

Despite some well-adapted clonal isolates, the herein described population is recombining. The evolutionary processes, sex crossing and consequently recombination, generates new combinations of genes, some of which may increase adaptation of the population to harsh environments to increase the chance of their survival [41]. On the other hand, DNA repair is a reasonable explanation for the high rate of recombination in diploid and haploid organisms, and could be an ancestral mechanism of general sexuality [46]. As recombination acts as ancient machinery of DNA repair, which is not only related to sexual reproduction, but also associated with a fast and simple way of propagation observed in the clonal reproduction, it is an advantage in overcoming the challenges of the environment [46, 47], with some of the well-adapted cells could become more virulent pathogens to humans (*e*.*g*. outbreak strains), as the killing of the host, in the case of an opportunistic *Cryptococcus* infection, will not interfere with fungal cell propagation.

Although the Brazilian isolates do not show a very well established population structure according to the geographic origin (Fig 4), we showed that the different Brazilian regions are dominated by different genotypes (Fig 1). The six sequence types identified (Fig 2) in more than one region may reflect the Brazilian human population migration patterns, *e*.*g*. as São Paulo and Rio de Janeiro (SE) are the biggest cities in the country, many people from other regions migrate to the these regions to find greater and better work possibilities. In order to check the influence of migration of *C*. *gattii* throughout the country, which could also be due to human migration, the clone corrected dataset was submitted to the admixture model in Structure and showed one basal population distributed all over the country (presented in red in Fig 4), one mainly found in the N and NE part of the country (presented in green in Fig 4), one more frequently found in the SE/CW region (presented in blue in Fig 4). Imported cases between these populations and within each State were also found (Fig 4).

The subtypes VGIIa and VGIIb, responsible for the outbreak on Vancouver Island, Canada [13, 34] and the subsequent spread to the Pacific Northwest of the USA [9, 40] have been identified in the North of Brazil (Fig 2). The sequence type ST20 (VGIIa) shows a large scattered distribution pattern in the Amazon region, with eight clinical isolates from the states of Pará, Amazonas and Roraima, and one environmental isolate from the state of Amazonas. In addition, three STs (ST122, ST266, ST267) linked to the clonal complex 20, mainly represented by ST20 (VGIIa), were also found in the Amazon region. The high frequency of this complex in the North may be related to a better adaptation/and or microevolution of these isolates to the environment, although one isolate of ST20 and the only isolate of ST252 were found in the city of São Paulo, which are most likely related to human migration processes. Imported cases caused by this sequence type have also been described in patients who had visited Vancouver Island from Denmark, Germany, Switzerland and the Netherlands [48, 49, 50] (presented in yellow in Fig 4).

The sequence type ST7 (VGIIb) has been found all over the world, including: Australia, Canada, China, Korea, Thailand and the USA [9, 17, 39, 45, 51, 52]. The Brazilian isolates of the sequence type ST7 (VGIIb) were found in the state of Amazonas. Besides these two outbreak associated sequence types, three additional sequence types from other countries have now been identified amongst Brazilian VGII strains, indicating further intercontinental spread as had been previously described [52, 53]. These include ST5, which had been reported from Australia [39], ST19, present in Greece [18, 54], and ST182, which has been found in France and China [55]. MLST analysis provided further evidence for close relationships between many Brazilian sequence types and the sequence types globally present (Fig 1). The sequence types ST7 (VGIIb), ST20 (VGIIa) and ST5, are the three sequences types identified in the current study which were also previously detected in dwelling dust samples and clinical specimens in the Amazonas state (North of Brazil), reinforcing the possibility of indoor infection, especially in wooden houses, very common in the northern part of Brazil, which was originally suggested by Brito-Santos *et al*. [56].

In the North and Northeast of Brazil, *C*. *gattii* behaves as an endemic fungal pathogen that causes infection in apparently healthy individuals categorized as immunocompetent patients, and the predominant VGII genotype has been recognized for at least the last 20 years among clinical and environmental strains from those large regions [57]. The results here reinforce recent findings supported by MLST and whole-genome SNP analysis indicating that the North American outbreak lineages, including the VGIIc genotype, which has only been found in the Pacific Northwest of the USA [12] but is closely related to South American strains [21], have most likely arisen from a highly recombining *C*. *gattii* population from South America, probably from the Amazon rainforest [12, 16, 18].

The detection of high genetic diversity amongst Brazilian *C*. *gattii* VGII isolates in the current study strongly supports the possibility of the emergence of highly virulent strains in the N and NE regions of Brazil, associated with different biotopes, one with extremely humid forest in the North (the Amazon Forest) and the other with open and predominantly dry savanna formations in Northeast (brushwood known as “caatinga”). Between them, there is a transitional region, with overlapping areas of humid forest, less humid tropical savannah (known as “cerrado”) then the dry caatinga, best observed in the states of Piauí and Maranhão.

An important finding of the current study is the central role of the ST278, which is associated with a clinical isolate (CFP 243) from the state Piauí and other closely associated STs from the same area. It shifts the global origin of *C*. *gattii* VGII, which was previously placed in the Amazon region in the state Roraima (CFP 439/LMM645 from 1998), North of Brazil, by Hagen *et al*. [18, 57] to the transitional ecological area in the Brazilian Northeast. Another very close lineage to ST278 is the ST124 from Piauí, isolated from clinical samples and decaying wood in tree hollows. One clinical sample was isolated from a case with cryptococcal meningitis and the other was isolated from the spleen of an armadillo without any evidence of disease. Thus, VGII has a potential wide host range, behaving as a multi-host pathogen. Protected microenvironments, such as tree hollows or armadillo burrows in Piauí state, probably play an important role in the *C*. *gattii* life cycle under such variable climatic conditions. These findings show the ecological adaptability of VGII to spread to new habitats, allowing it to survive in dry and humid warm or cold climate.

The Brazilian Northeast and North are large geographical regions, which have been subject to extensive deforestation, leading to enormous landscape changes and the establishment of new settlements, disturbing original communities and related habitats, and causing a large-scale biodiversity loss. These habitat changes, shifts in species composition and other stress factors may affect the profile of *C*. *gattii* populations, inducing recombination events (and/or hybridization). Human-induced land use and extensive trade of native wood from the Amazon rainforest are also possible drivers of geographical dispersal of propagules, and consequently, disease emergence events.

Taking into account that the present study has, like all previous studies, a possible sampling bias, over-representing some STs while others are underrepresented, it is necessary to suggest further studies investigating other ecological niches, such as a variety of human inhabited places as well as environmental samples from other tropical countries.

The results indicate that the isolates from the transitional ecological area in Northeast Brazil are the most likely ancestor lineages, translocating from caatinga/cerrado by adapting progressively throughout Amazonia in South America, and spread to the North American Pacific Northwest regions and other parts of the world on multiple occasions. This picture is intrinsically related to climatic changes and devastating human activities globally. Therefore, a multifocal origin for the outbreak lineages of cryptococcal infections must be considered.

## Supporting Information

S1 Table Data of 194 strains analysed in the study. (XLSX)
